# Supplementary material for: Impact of quarantine due to COVID-19 pandemic on health and lifestyle conditions in older adults from Centro American countries
Source: PLoS One. 2022 May 11;17(5):e0264087. doi: 10.1371/journal.pone.0264087 (PMC9094529; doi:10.1371/journal.pone.0264087)
Supplement: Attachment — Submitted filename: Response to reviewers.docx [file pone.0264087.s001.docx]

Response to reviewers

Dear reviewers we appreciated your comments to our paper, there were incredible valuable to improve the paper robustness and clarify our ideas about the topic.

Reviewer 1

Minor comments:
1) As the authors rightly pointed out “The present study must acknowledge some limitations” and have acknowledged few limitations in their study. So, to make this study more general the author should increase the sample size. The presented sample set of 712 is too small in number. If the authors were unable to increase the sample size, then, they should acknowledge this limitation in their manuscript.

We added the following text to clarify that limitation:

“Another limitation is that due to the sampling strategy and sample size, representativeness is not guaranteed, although the inclusion of older adults from five different countries includes diversity and heterogeneity in sampling”.

2) Considering the socio-economic conditions, it’s not clear to me how strict was the lockdown in the Centro American countries and how strongly the subjects adhered to these quarantine measures.

We added the following text to clarify your observation, regarding the pandemic restrictions:

For this reason, the adoption of quarantine measures began in March 2020 except in Mexico where toughened measures were applied since May. In pandemic early stages, the level of compliance was quite high, associated with the closure of non-essential activities, closure of borders and even the imposition of fines on those who did not comply with the quarantine measures. However, citizens began to experience "pandemic fatigue" because of restrictive measures and the need to contribute with economic income to their families (34)

3) Also, I am curious if the authors have any data about the weight loss or weight gain during this time.

Unfortunately, we do not have that data.

Reviewer 2

1. The language of manuscript may be polished (minor).

The manuscript was reviewed by native English speaker to assure the quality of language.

2. The abbreviations should be cross validated in the manuscript (First define them fully followed by abbreviation).

We have completed your suggestion and were highlighted in the manuscript.

3. Please provide few information’s about health and lifestyle conditions in older adults before COVID-19 Pandemic in discussion (minor).

We added the following text to tackle your observation:

In years prior to the pandemic, a progressive higher prevalence of non-communicable diseases was observed. Cardiovascular diseases were the more prevalent and were associated with excessive consumption of alcohol, tobacco, sedentarism and unhealthy diet (25).

4. The authors may additionally provide one Figure as summary, challenges, or prospect of the present study.

We consider that information in discussion is covering this observation, mostly about challenges and prospect of the study.
